# Data on the draft genome sequence of *Caryocar brasiliense* Camb. (Caryocaraceae): An important genetic resource from Brazilian savannas

**DOI:** 10.1016/j.dib.2019.104543

**Published:** 2019-09-23

**Authors:** Rhewter Nunes, Ariany Rosa Gonçalves, Mariana Pires de Campos Telles

**Affiliations:** a Laboratório de Genética & Biodiversidade, Instituto de Ciências Biológicas - UFG, Goiânia, 74690-900, Brazil; b Escola de Ciências Agrárias e Biológicas, PUC-GO, Goiânia, Brazil

**Keywords:** Cerrado, Genome assembly, Genome evolution, Native plants, SSR markers

## Abstract

*Caryocar brasiliense* (Caryocaraceae) is a Neotropical tree species widely distributed in Brazilian savannas. This species is very popular in central Brazil mainly due to the use of its fruits in the local cuisine and their anti-inflammatory proprieties, and indeed it is one of the candidates, among Brazilian native plants, for fast track incorporation into cropping systems. Considering the importance of *Caryocar brasiliense*, little is known about its genetics and genomics, and determination of a reference genome sequence could improve the understanding of its evolution, as well as the development of tools for domestication. Here, we provide the first draft genome of *C. brasiliense*, the raw sequencing data and some multiplex sets of high quality microsatellite primers. Data on the genome project can be obtained from the BioProject at NCBI (https://www.ncbi.nlm.nih.gov/bioproject/?term=caryocar).

**Table:** Specifications Table

Subject area	Biology
More specific subject area	Genomics, horticultural science.
Type of data	Whole genome sequence data, genome assembly and primers for candidate microsatellites markers.
How data were acquired	High-throughput sequencing (Illumina HiSeq 2000).
Data format	Raw sequencing reads (fastq) and draft-genome (fasta).
Experimental factors	Sequencing was performed using Illumina HiSeq, and the draft genome was determined using Platanus software.
Experimental features	Sequencing was performed according to Illumina Nextera protocol for DNA-Seq.
Data source location	Agronomy School, Federal University of Goiás - Goiânia, Goiás, Brazil (16°35′49.8″S 49°16′45.4″W).
Data accessibility	The complete genome sequence of *Caryocar brasiliense* is available in the NCBI GenBank under accession number: STGP00000000. The sequencing reads used in assembly analysis are available in the NCBI SRA database under accession number: SRX5692978 (https://www.ncbi.nlm.nih.gov/sra/?term= SRX5692978).

**Table:** 

**Value of the Data** • This dataset provides the first version of a draft genome for *Caryocar brasiliense*. This is the first genome project for a species from the Caryocaraceae family and can be used as a reference in future genome projects for other species. • This dataset can be used for comparative analyses in evolutionary studies. The draft genome can be used to identify genes, repeat regions, microsatellites and other genome elements that can describe the biology and evolution of the species. • Primer data can be used for the development of molecular markers for domestication and breeding programs. We selected and made available some high quality multiplex microsatellite sets for genetic diversity analysis.

## 1 Data

The pequi (*Caryocar brasiliense* Camb.) belongs to the family Caryocaraceae (Malpighiales order) and is an important genetic resource from Brazilian savannas mainly because of the use of its fruits in local cuisine and their anti-inflammatory proprieties. We present the first draft genome of *C. brasiliense* using high-throughput DNA sequencing, the raw sequencing data used in the genome assembly analysis and a set of primers to amplify candidate microsatellite markers. The draft genome recovered 45.69% of the estimated genome size (464,365,380 bp) distributed in 55,248 contigs (Table 1). The draft genome is available at: https://www.ncbi.nlm.nih.gov/nuccore/STGP00000000.1/. The raw reads dataset was obtained from a run using Illumina HiSeq2000 equipment. A total of 293,621,819 sequencing reads of 100 base pairs each were generated. Sequencing data are available at: https://www.ncbi.nlm.nih.gov/sra/?term= SRX5692978. Additionally, 5 multiplex with 5 to 7 high-quality microsatellite primers (total of 30 pairs of primers) were designed and are available in this paper (Table 2).

**Table 1:** Genome assembly statistics of the draft genome of *Caryocar brasiliense*.

Metric	Value
Number of contigs	55,248
Number of contigs ≥1000 bp	43,286
Total length	212,172,521
Largest contig	64,707
Shortest contig	500
N50	6005
N75	3615
L50	10,532
L75	21,784
GC%	34.84

**Table 2:** Multiplex microsatellite primers designed for *Caryocar brasiliense*.

Multiplex_ID	Primer_ID	SSR_Motif	Primer_Foward_5′-3′	Primer_reverse_5′_3′	Ta	PCR_Frag_len
1	Cbr_NGS_SSR1	TATG	gctacttccagtcactagacttgt	cacaactgctaccatgttcgac	62	349
1	Cbr_NGS_SSR2	CATA	acccgccttctccagtgaata	tcctcgagttttacagcggtat	60	164
1	Cbr_NGS_SSR3	CT	ctctctttgcgggatatctcaaga	ccatgacagtccagcccaata	61	224
1	Cbr_NGS_SSR4	CT	actctgccgacagctgaattta	aaaggcaacacagcagatcattaa	60	102
1	Cbr_NGS_SSR5	AG	gtggaaatgcataaactgtatgcct	cgatagctgctcttgccaagt	62	584
1	Cbr_NGS_SSR6	TC	gcttctgcaaaatcataggcaaca	agtggtaattcacgctggtaattta	60	425
1	Cbr_NGS_SSR7	TTC	gccattctcaattttccagtggac	gtgtgtgttgtaaacattcaaggat	60	493
2	Cbr_NGS_SSR8	AGG	aataagatgccattgcggtgtt	tgaccgactctttcttattgggaa	60	157
2	Cbr_NGS_SSR9	TC	tacataaattgtcttcagcccatgt	agcctgctcgattaagtgaaca	60	278
2	Cbr_NGS_SSR10	GCA	agagtccttgtgacgaatcagatt	ctcatccgagaacttatgcagc	60	218
2	Cbr_NGS_SSR11	GAT	gccatcagcgaacagttctct	caacaaattacctgctccgagtt	61	372
2	Cbr_NGS_SSR12	TTC	gagttttgatgcttaagccatgac	gccttaccagagtctgcaagt	61	434
2	Cbr_NGS_SSR13	GGT	ccactgacttattcaatttctcgac	ggaccctcaacaggacctattt	60	513
3	Cbr_NGS_SSR14	AG	gaactcttttccctacagatcagaa	catttcaggttgagtagcttgtca	60	270
3	Cbr_NGS_SSR15	GCT	ggacgccatttcacaagattga	ccctgctgtcaacaggattct	61	132
3	Cbr_NGS_SSR16	CTT	aggatgcctttccaaagacgt	ttttacagcaacatttgtgagactc	60	331
3	Cbr_NGS_SSR17	CAA	ttaatgatctggggtcacatcctt	gtgggggcaatggacctaatat	60	195
3	Cbr_NGS_SSR18	GTT	ggagatcagaccaagcattgct	tgcatcattttggcgactacaat	61	495
3	Cbr_NGS_SSR19	TTC	gaggctgcattaagcatggaaa	aagacaaaagagtggatttcccac	61	402
4	Cbr_NGS_SSR20	GAA	aaaactggtagaagatgcagtcaa	gattagaatgtgcaaaattggcagt	60	312
4	Cbr_NGS_SSR21	CTT	aacggggtcccatcgtatctt	gacacctgttaagcaagaacatgt	62	251
4	Cbr_NGS_SSR22	CTT	cggtatatggaagcgtacttcac	tctgcactcgcaagtccaata	60	176
4	Cbr_NGS_SSR23	GTT	gcttttgttgtggagccaaattaca	cgcgaaattcctcatgttcaga	60	109
4	Cbr_NGS_SSR24	GTT	gtcattaacctgacaccattgct	tctactgctatgttcggagcatatt	61	392
5	Cbr_NGS_SSR25	GA	tattcaggcgtggcaccaata	tggctcaaaactttgcatactgat	61	258
5	Cbr_NGS_SSR26	GA	ctgcttcagttcggagaccaa	atctacttccaaagacatagtgtgc	61	332
5	Cbr_NGS_SSR27	GA	cgtcaaatcttccaacagctga	catgtttcattgaagggccatcat	60	180
5	Cbr_NGS_SSR28	CT	aggtgatgtgaccttccaagc	agaatggggattcgtgttctagtt	61	447
5	Cbr_NGS_SSR29	GA	ctagcagtgcttcgtcaaaactt	ttattcagtgacccggttatggat	60	111
5	Cbr_NGS_SSR30	TC	gttcagcaaacattctgctaagtc	ttgggaacgtaaagatcaatttcct	60	508

## 2 Experimental design, materials, and methods

### 2.1 Total DNA sampling and sequencing

Fresh leaves were collected from a tree at Escola de Agronomia, Universidade Federal de Goiás, Goiânia, Goiás, Brazil (16°35′49.8″S 49°16′45.4″W). The total DNA was extracted from leaves using the CTAB protocol [1]. The quality of DNA was determined by a Nanodrop device, and the quantity was measured by a Qbit and 1% agarose gel. The sample was sent to Centro de Genômica Funcional ESALQ-USP core facility for sequencing. An Illumina paired-end 2 × 100 bp library was constructed and forwarded for sequencing using an Illumina HiSeq2000 platform.

### 2.2 Sequencing quality control and assembly

Raw reads were evaluated for base quality sequencing and sequencing adapter presence using FastQC software (http://www.bioinformatics.babraham.ac.uk/projects/fastqc/). Quality control was performed using Trimmomatic software v0.39 [2] with the options ILLUMINACLIP: TruSeq3-PE.fa:2:30:10 and SLIDEWINDOW: 4:30, which required at least a mean Phred score of 30 for every four bases. The best k-mer value was estimated using Kmergenie software [3]. The *de novo* assembly was performed using Platanus (PLATform for Assembling NUcleotide Sequences) software v1.2.4 [4].

### 2.3 Microsatellite identification and primer design

The microsatellite regions were identified in the genome using QDD software [5]. The program marks the primers for microsatellite regions that occur in the context of transposable elements. This allows the selection of the best primer pairs for the molecular marker test as it minimizes the occurrence of null alleles due to primer annealing problems. We used only contigs larger than 10 Kb in the microsatellite analysis. After identification of the microsatellite regions, we applied a rigorous filter to choose the best sets of primers for molecular marker tests. Among the 120,858 pairs of primers designed for 6885 identified microsatellite regions we applied the following filters: i) primers with a size between 20 and 24 base pairs; ii) PCR product size between 150 and 460 base pairs; iii) not including a region formed only by adenine and thymine bases; iv) at least 16 dinucleotide, 6 trinucleotide, 6 tetranucleotide and 4 pentanucleotide repeats and v) the difference in annealing temperature between the primers is less than 2 °C. For the resulting set of primers, the best pair for each microsatellite region was chosen based on the greatest possible distance between target regions and primers. We used FastPCR software to generate the multiplex sets [6]. The final set of primers we recommend for testing as molecular markers correspond to 30 microsatellite regions distributed in a set of 5 PCR multiplex.
